# Advanced bipolar vessel sealing devices vs conventional bipolar energy in minimally invasive hysterectomy: a systematic review and meta-analysis

**DOI:** 10.1007/s00404-023-07270-8

**Published:** 2023-11-13

**Authors:** Pier Carlo Zorzato, Filippo Alberto Ferrari, Simone Garzon, Massimo Franchi, Stefano Cianci, Antonio Simone Laganà, Vito Chiantera, Jvan Casarin, Fabio Ghezzi, Stefano Uccella

**Affiliations:** 1 Unit of Obstetrics and Gynecology, Department of Surgery, Dentistry, Pediatrics, and Gynecology, University of Verona, AOUI Verona, Verona, Italy; 2 Department of Obstetrics and Gynecology, University of Messina, Messina, Italy; 3 Department of Health Promotion, Mother and Child Care, Internal Medicine and Medical Specialties (PROMISE), University of Palermo, Palermo, Italy; 4 Department of Obstetrics and Gynecology, Filippo Del Ponte Hospital, University of Insubria, Varese, Italy; 5 Unit of Obstetrics and Gynecology, Paolo Giaccone Hospital, Palermo, Italy; 6 Unit of Gynecologic Oncology, National Cancer Institute - IRCCS - Fondazione “G. Pascale”, Naples, Italy

**Keywords:** Advanced bipolar vessel sealing device, Conventional bipolar instrument, Energy, Laparoscopic hysterectomy

## Abstract

**Purpose:**

To compare conventional bipolar electrosurgery with advanced bipolar vessel sealing (ABVS) devices for total laparoscopic hysterectomy (TLH).

**Methods:**

A systematic review was conducted by searching Scopus, PubMed/MEDLINE, ScienceDirect, and Cochrane Library from January 1989 to November 2021. We identified all studies comparing ABVS devices with conventional bipolar electrosurgery in TLH and reporting at least one of the following outcomes: total blood loss, total operative time, hospital stay, perioperative complications, or costs. Meta-analysis was conducted with a random effect model reporting pooled mean differences and odds ratios (ORs) with related 95% confidence intervals (CIs).

**Results:**

Two randomized controlled trials and two retrospective studies encompassing 314 patients were included out of 615 manuscripts. The pooled estimated total blood loss in the ABVS devices group was lower than conventional bipolar electrosurgery of 39 mL (95% CI − 65.8 to − 12.6 mL; *p* = .004). The use of ABVS devices significantly reduced the total operative time by 8 min (95% CI − 16.7 to − 0.8 min; *p* = .033). Hospital stay length did not differ between the two groups, and a comparable overall surgical complication rate was observed [OR of 0.9 (95% CI 0.256 – 3.200; *p* = .878].

**Conclusions:**

High-quality evidence comparing ABVS devices with conventional bipolar electrosurgery for TLH is lacking. ABVS devices were associated with reduced total blood loss and operative time; however, observed differences seem clinically irrelevant. Further research is required to clarify the advantages of ABVS devices over conventional bipolar electrosurgery and to identify cases that may benefit more from their use.

## Introduction

Hysterectomy is the eighth most common surgical procedure and the most performed gynecological surgery in Europe [1]. The minimally invasive approach by laparoscopy is widely feasible and guarantees an earlier return to normal activities, reduced hospital stay, and higher patient satisfaction compared to the open technique [2]. However, large uteri, previous surgeries, distorted pelvic anatomy, and other technical limitations could lead to complex procedures with an increased risk of conversion to laparotomy or urinary tract and bowel injuries [3–5]. In this scenario, tissue dissection, transection, and hemostasis may be simplified and made safer by the application of newly developed energy sources and instruments able to widen the application of the minimally invasive approach.

The first applied monopolar instrument was a low-cost and widely available option. Still, its use has progressively decreased, favoring conventional bipolar instruments, which have been developed to reduce energy-related injuries and to provide more efficient vessel coagulation [6]: the electricity flows between the grasper jaws delimiting the thermal impact in the proximity of the electrodes [7]. However, the major drawbacks of conventional bipolar instruments are the surgeon-dependent compression of tissues and duration of activation, which may determine incomplete vascular occlusion and the risk of lateral thermal damage.

Advanced bipolar vessel sealing (ABVS) devices have been implemented to overcome these limitations. These instruments operate with high pulsatile current and lower voltage energy, allowing tissue cooling during activation, limited thermal spread, and adequate tissue compression [8]. While conventional bipolar instruments obtain coagulation by determining the formation of thrombi in the vessels, ABVS instruments generate an actual sealing of arteries and veins, thus providing safer hemostasis. Furthermore, most ABVS devices have a computer-assisted tissue feedback response, which monitors tissue impedance to guarantee adequate tissue sealing, and an integrated cutting system allows cutting without additional instruments. Several devices are available on the market with different designs, mechanical systems, or tissue-impedance monitoring technologies, but all have similar performance and are approved for sealing vessels up to 7 mm in diameter [9]. In the last years, the use of ABVS devices and their research interest significantly increased in all surgical specialties [10], and the advantages, as mentioned above, seemed to balance or overcome the high costs [11, 12].

However, the surgical procedure highly influences the pros and cons of ABVS devices. Results of studies on complex general surgical [13–15] or urological [16–18] operations may not be generalizable to other surgical procedures. For these reasons, we performed this systematic review and meta-analysis to summarize evidence comparing ABVS devices with conventional bipolar electrosurgery instruments in total laparoscopic hysterectomy (TLH) to clarify differences and possible advantages in clinical practice.

## Methods

Before starting the online search, the research protocol was developed, considering research questions, populations of interest, outcome measures, search strategies, study eligibility criteria, and planned analyses, including subgroup analyses. This protocol has been registered in the International Prospective Register of Systematic Reviews (PROSPERO: CRD42021279245) and was deemed exempt from institutional review board approval.

### Search strategies

A certified professional librarian (Biblioteca Meneghetti—University of Verona) performed a literature search from January 1989 to November 2021 in the electronic databases Scopus, PubMed/MEDLINE, ScienceDirect, and the Cochrane Library. The search strategy included the combinations of the Medical terms “Hysterectomy,” “Hysterectomy, Vaginal,” “Laparoscopic hysterectomy,” “Advanced vessel sealing device,” “Advanced bipolar energy device,” “Bipolar vessel sealing,” “Vessel sealing,” “Reusable energy devices,” “Single-use energy device,” “Conventional bipolar instrument,” “Conventional bipolar electrosurgery,” “Ultrasonic Energy,” “Harmonic energy,” “Energy devices,” “Bipolar electrosurgery,” “EnSeal,” “Gyrus,” “LigaSure,” and “Thunderbeat.” The references of all identified studies were systematically revised to identify other eligible publications.

### Inclusion and exclusion criteria

We included all full-text manuscripts published in the English language selected based on pre-specified PICO criteria. Population: women who underwent TLH with or without bilateral adnexectomy for benign gynecological pathology; Intervention: hysterectomy performed with ABVS devices; comparison: hysterectomy performed with conventional bipolar instruments; outcomes: estimated blood loss, total operative time (from skin incision to skin closure), complications rate (visceral injury, including bladder, bowel, and ureteral damages; significant blood loss requiring conversion to laparotomy, reoperation, readmission for pelvic infection), hospital stay length, and estimated costs. Outcome eligibility required reporting at least one of the outcomes of interest. Regarding the study design, we included both randomized controlled trials (RCT) and observational studies (whether prospective or retrospective).

From identified publications, we excluded abstracts, brief reports, or congress proceedings. Moreover, we excluded studies, including less than 15 patients per arm, hysterectomies performed for malignant disease, supracervical hysterectomy, hysterectomies performed by open or vaginal approaches, and laparoscopic-assisted vaginal hysterectomy.

### Study selection and data extraction

Titles and abstracts of identified studies by the initial literature search were screened independently by two authors (FAF, PCZ). The full text of the potentially eligible studies was retrieved and independently assessed for eligibility by other review team members (SG, SU). Any disagreement over the eligibility of studies was resolved through discussion with a further author (MF). A standardized form was developed and used to extract data from eligible studies: characteristics of trial participants (including age, diagnosis, and the number of patients per arm), type of intervention (ABVS device and conventional bipolar instrument details, such as brand and model), and outcomes measures with details regarding their assessment and used definition (total blood loss and mode of quantification, total operative time, hospital stay length, costs of the procedure, readmission up to 30 days, and the number of patients reporting at least one complication during and up to 30 days after surgery). One review author (FAF) extracted the data from included studies, and a second author (PCZ) checked the extracted data. Disagreements were resolved by discussion between the two review authors; if no agreement was reached, a third author (SG) decided. The review and metanalysis were written following the Preferred Reporting Items for Systematic Reviews and Meta-Analyses (PRISMA) guidelines [19].

### Quality assessment

Two review authors (FAF, PCZ) independently assessed the risk of bias in included studies according to the Cochrane tool, separating RCTs from non-RCTs [20].

### Strategy for data synthesis

The meta-analysis was performed using a random-effects model (instead of fixed-effects), since we could not assume that all studies had a common treatment effect. We did not expect a common treatment effect for all included studies but rather that the variation of the effects across studies follows the same distribution. Included studies did not have the same population, the same surgeon, and the same instruments; therefore, both within- and between-study variability must be considered [21–23]. Pooled mean difference and 95% confidence intervals (CIs) were estimated for continuous variables. Pooled odds ratios (ORs) with 95% CIs were used for categorical variables. Heterogeneity was tested using the I^2^ tests; I^2^ less than 25% was considered low, and I^2^ more than 75% was deemed to be high. All analyses were two-tailed with a statistical significance threshold of *p* = 0.05. Open Meta version 5 was used to conduct meta-analyses.

## Results

**Fig. 1:**
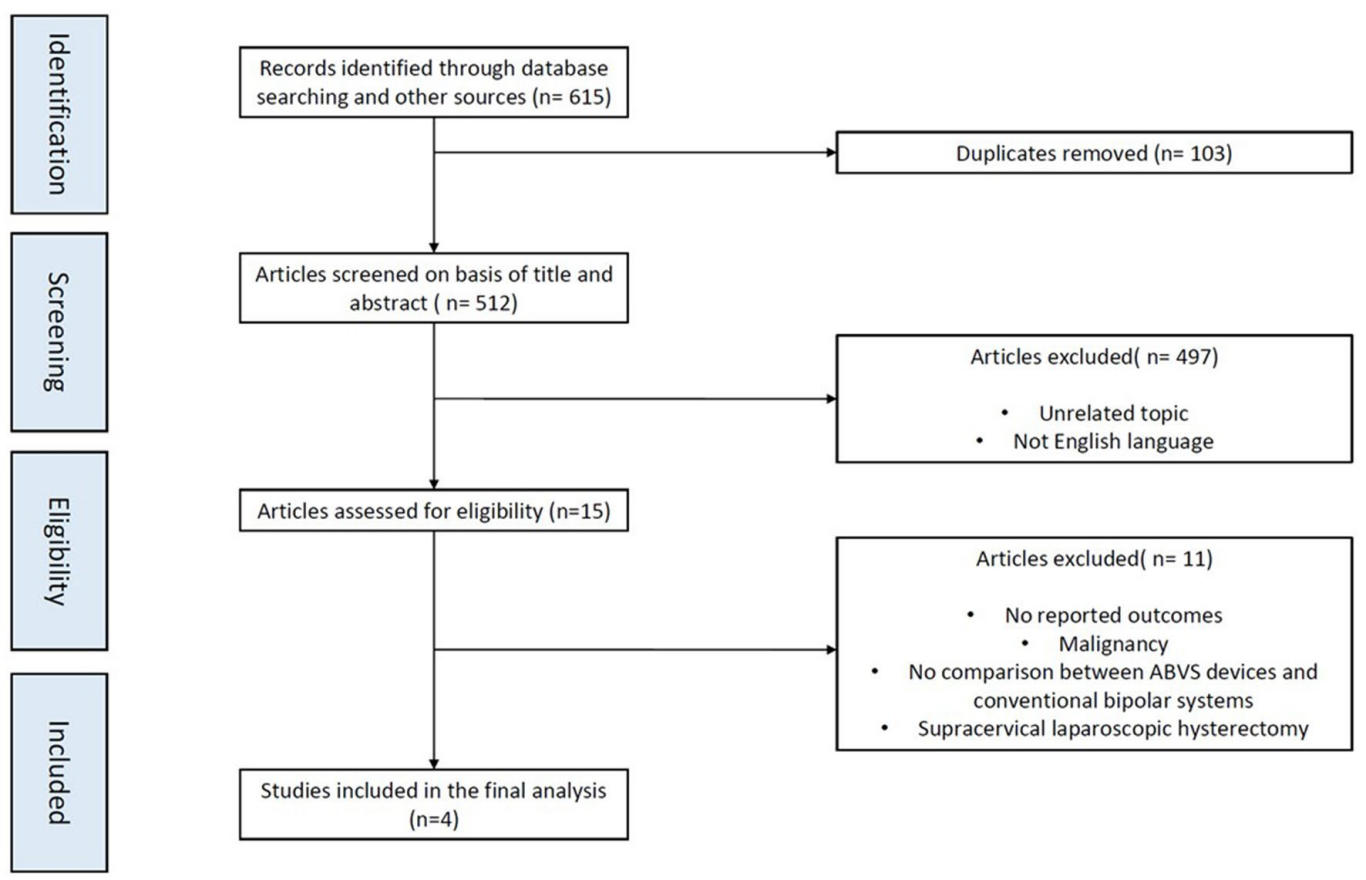
Flow diagram of study selection

Our literature search identified 615 papers, including studies identified with cross-reference review. Duplicates were excluded, and after the title and abstract screening, 15 potentially relevant articles were identified and underwent full-text assessment for eligibility. One study [24] was excluded from 15 studies [5, 6, 24–36], because it did not report data on relevant outcomes. Seven studies did not compare ABVS devices with conventional bipolar systems [6, 25, 27, 28, 31, 33, 34], two studies included hysterectomies for malignant indication [35, 36]. One study reported supracervical laparoscopic hysterectomies [32]. The flowchart of article selection for the meta-analysis is summarized in Fig. 1.

A total of 4 studies (two RCTs and two non-RCT studies) were finally included in the systematic review and meta-analysis (Table 1), with a total of 151 and 163 patients in the ABVS device and conventional bipolar arms, respectively. Janssen et al. enrolled patients who underwent total laparoscopic hysterectomy or supracervical laparoscopic hysterectomy and only the first group was included in the pooled analysis [5]. No study comparing ABVS devices and conventional bipolar electrosurgery instruments during robotic hysterectomy was found.

**Table 1:** Characteristics of the studies included in the present meta-analysis

Author	Study design	Energy devices in TLH	N. Patients included	Blood loss (*p* value)	Total operative time (*p* value)	Hospital stay (*p* value)	Total
Ou [30]	Retrospective	Conventional bipolar vs PKS	46 vs 37	225.0 ± 195.0 mL vs 140.0 ± 45.9 mL (p = *.034*)	68.6 ± 15.9 min vs 69.2 ± 15.9 min (p = *.925*)	NA	83
Janssen et al. [5]	RCT	Conventional bipolar vs Ligasure	53 vs 57	305.9 ± 375 mL vs 232.6 ± 286 mL (*p* = *.249*)	147.2 ± 48.7 min vs 140.3 ± 39.0 min (*p* = *.412*)	2.9 ± 1.1 d vs 2.8 ± 0.8 d (NA)	110
Cho et al. [26]	Retrospective	Conventional bipolar vs PKS	40 vs 40	515.3 ± 41.2 mL vs 467.9 ± 33.4 mL (*p* = .05)	173.4 ± 33.4 min vs. 157.3 ± 21.3 min (*p* = .001)	6.5 ± 1.3 d vs 6.2 ± 1.2 d (NA)	80
Lee et al. [29]	RCT	Conventional bipolar vs Ligasure	35 vs 36	310.60 ± 220.60 mL vs 269.23 ± 232.47 mL (*p* = .445)	99.54 ± 31.96 min vs 85.58 ± 30.21 min (*p* = .063)	3.37 ± 0.77 d vs 3.34 ± 0.54 d (*p* = .858)	71

mL: milliliters; min: minutes; d: days; TLH: total laparoscopic hysterectomy; PKS: plasma kinetic system; NA: not available; RCT: randomized controlled trials

Studies investigated only two ABVS devices: Ligasure (Covidien, Mansfield, MD) was used in 94 patients, and Plasma Kinetic system (PKS; Gyrus ACMI, Southborough, MA) in 57 cases. In the conventional bipolar group, the Kleppinger forceps (Richard Wolf Instruments, Vernon Hills, Illinois) were used in 86 patients [26, 37], and the Eragon Grasping and Dissecting Forceps Maryland Dissector 5 mm (GmbH, Knittlingen, Germany) in 35 cases [35], and Seitzinger or a Cutting forceps (both formerly produced by ACMI Corp., Southborough, MA, USA) in 42 patients [5].

The included studies provided enough data to allow the pooled analysis for intraoperative total blood loss, total operative time, hospital stay length, and complication rate. Conversely, cost analyses were provided by one study for Ligasure [5] and by one study for PKS Gyrus [37]; therefore, the reported data were inadequate to perform a meta-analysis.

### Blood loss

**Fig. 2:**
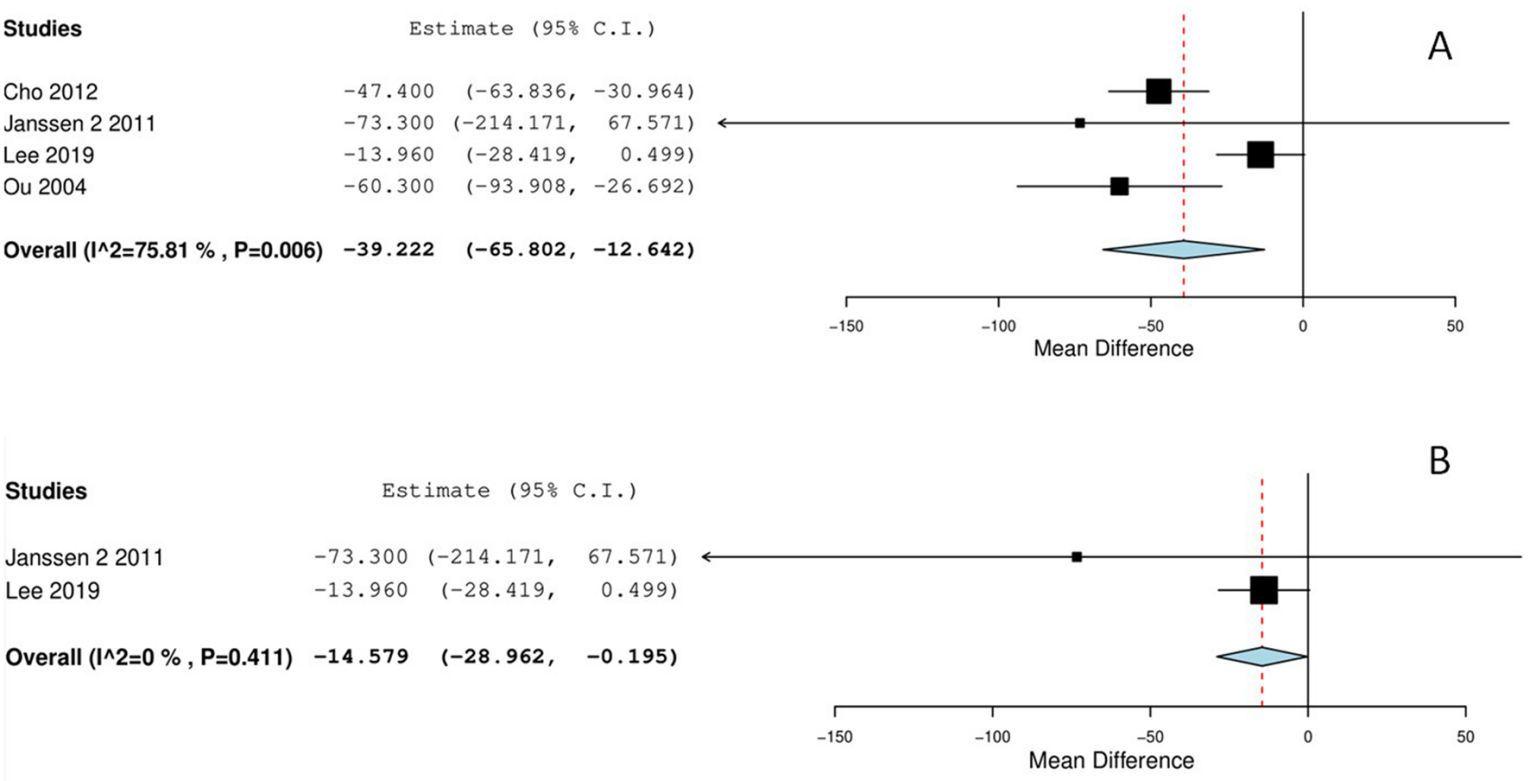
**a** Intraoperative blood loss; ABVS device vs. conventional bipolar electrosurgery. **b** Intraoperative blood loss in randomized trials; ABVS device vs. conventional bipolar electrosurgery

All included studies reported the average intraoperative estimated blood loss and relative standard deviations for the two groups. Two studies did not report the used estimation method [26, 37]; the others estimated blood losses from the swab, gauze, and suction bags by subtracting irrigation fluids [5, 29]. In the pooled analysis, the average total intraoperative blood loss was lower in the ABVS devices group than in the conventional bipolar systems by -39 mL (95% CI  -65.8 to -12.6 mL; *p* = 0.004; Fig. 2a). However, the heterogeneity among trials was high, with *I*^2^ = 75%, *p* = 0.006, Fig. 2a). In the sub-analysis, including only RCT trials, a lower total intraoperative estimated blood loss with ABVS devices was confirmed (-14.6 mL; *p* = 0.047; 95% CI  -28.9 to -0.2 mL; Fig. 2b), although one study by Lee et al. weighted for 98% of the total.

### Total operative time

**Fig. 3:**
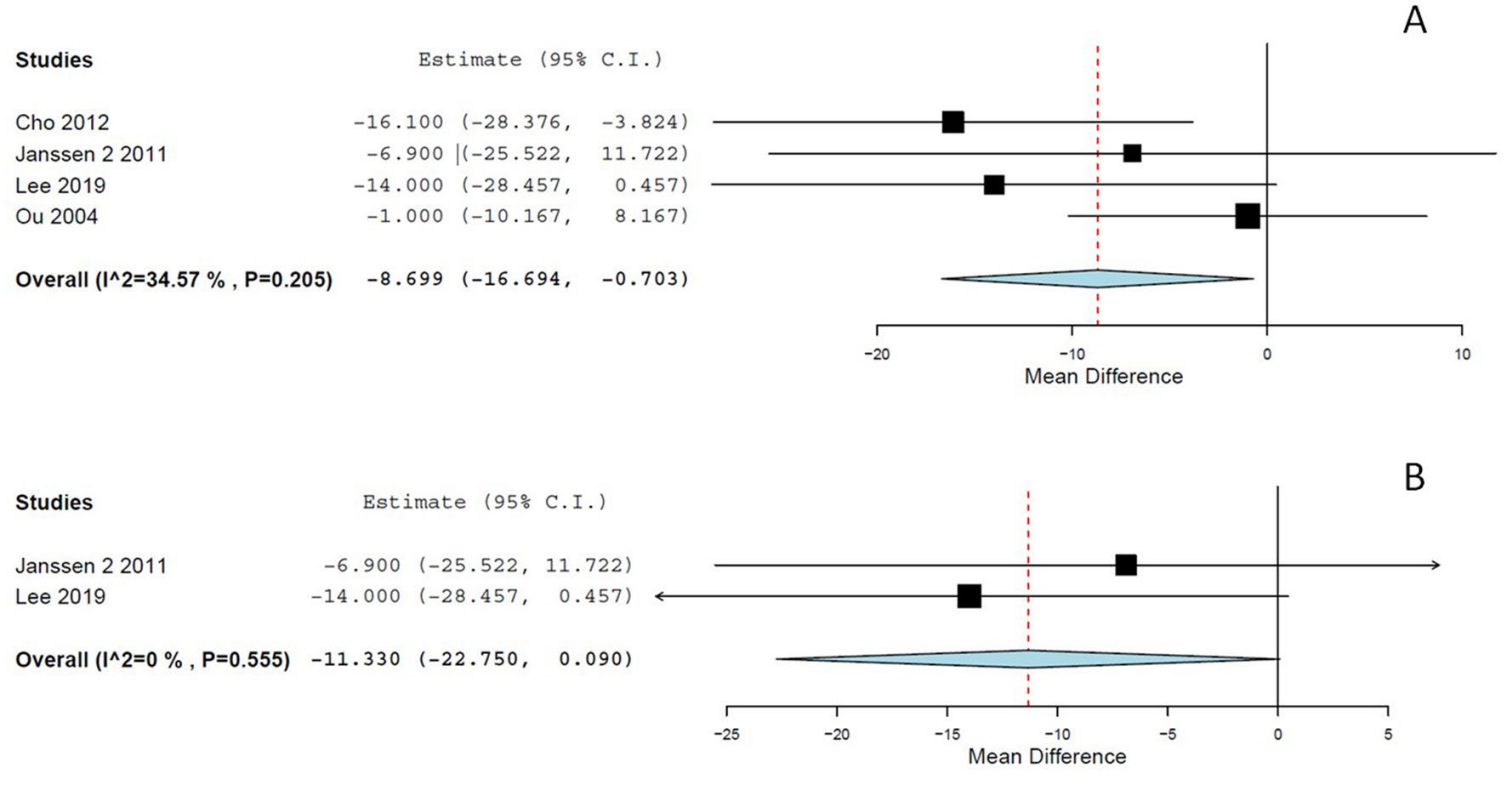
**a** Total operative time; ABVS vs. conventional bipolar electrosurgery. **b** Total operative time in randomized trials; ABVS vs. conventional bipolar electrosurgery

Two studies provided partial operative time [5, 35], and all included studies reported total operative time using the same definition from skin incision to skin closure. We included only the total operative time in the meta-analysis per the pre-specified study protocol. The meta-analysis revealed that the use of ABVS devices reduced the whole operative time by -8.7 min (95% CI  -16.7 to -0.7 min; *p* = 0.033; Fig. 3a) with moderate heterogeneity (*p* = 0.205, *I*^2^ = 34.57%, Fig. 3a). However, the pooled analysis including only RCTs did not show a statistically significant difference of -11.33 min (95% CI  -22.75 to 0.09; *p* = 0.052; *p = 0.555,* *I*^2^ = 0%; Fig. 3b).

### Hospital stay length

**Fig. 4:**
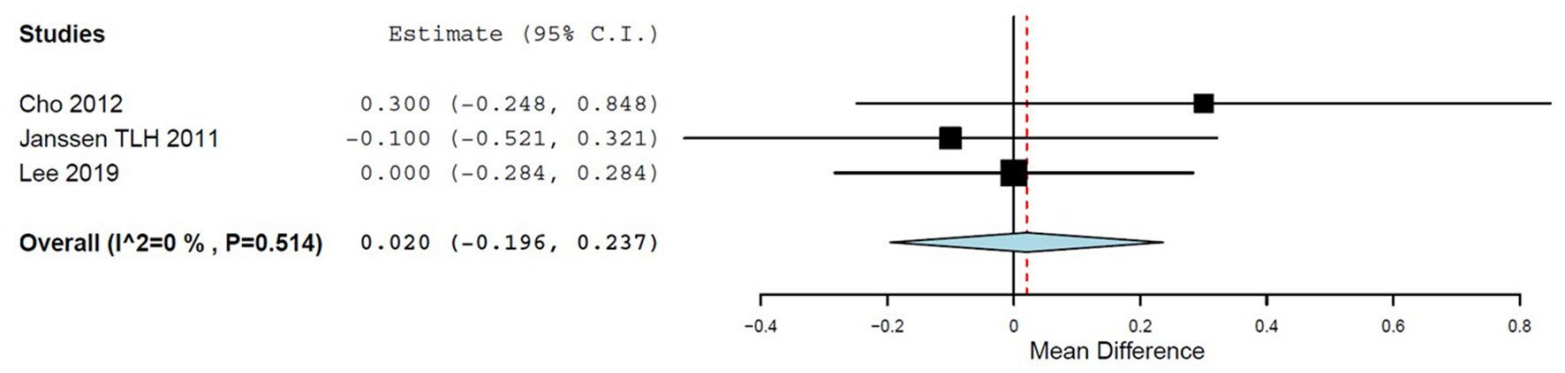
Hospital stay length; ABVS vs. conventional bipolar electrosurgery

All but one study provided data about the hospital stay length [37]. The pooled analysis showed no statistically significant differences between conventional bipolar systems and ABVS devices (*p* = 0.85; 95% CI  -0.196 to 0.237; *p = 0.514, I*^2^ = 0%; Fig. 4), even including only RCTs (*p* = 0.79).

### Complications

None of the included studies reported intraoperative complications attributable to ABVS devices or conventional bipolar systems. The definition of complications was heterogeneous, and none of the included studies followed a standardized classification to attribute the severity. The length of follow-up and items considered were various, making the analysis inconclusive on the real impact of ABVS devices. All but one study provided intraoperative or post-operative complications data in the considered groups [5]. In both the traditional bipolar and ABVS devices groups, a total of 5 intraoperative complications per arm were observed in the pooled analysis, with an incidence of 4.1% and 4.7% in conventional bipolar instruments and ABVS devices groups, respectively (OR of 0.9; 95% CI 0.256 to 3.200; *p* = 0.878).

### Risk of bias assessment

**Fig. 5:**
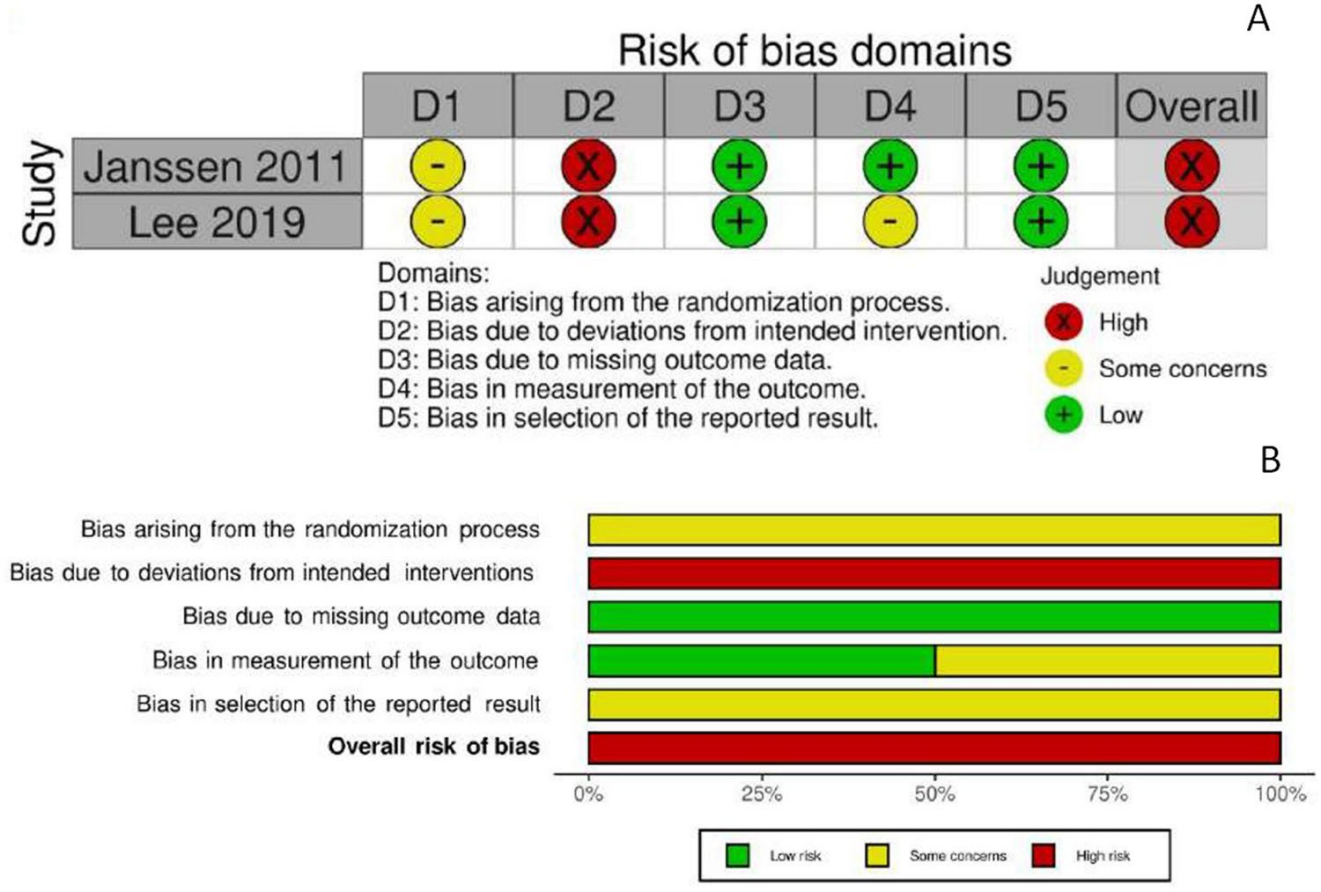
**a** Risk of bias, randomized trials. **b** Overall risk of bias, randomized trials

**Fig. 6:**
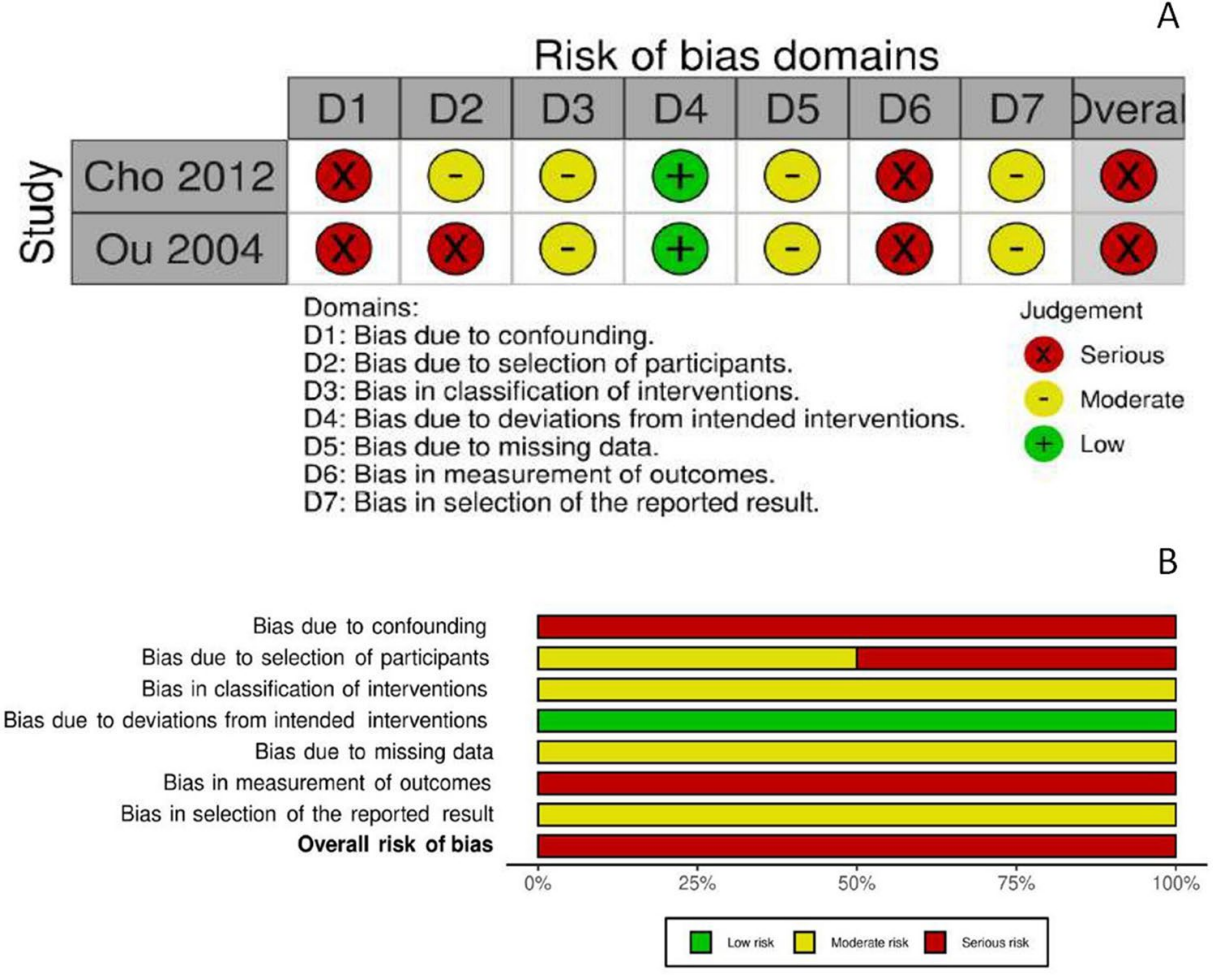
**a** Risk of bias, non-randomized trials. **b** Overall risk of bias, non-randomized trials

The risk of bias assessment is summarized in Fig. 5a and 5b for RCTs, and in Fig. 6a and 6b for non-RCTs studies. The two RCTs (100%) had a severe risk of bias. Similarly, non-RCTs were estimated at high risk of bias. All included studies lacked blinding, given the surgeon was necessarily aware of the surgical device during surgery.

## Discussion

In this systematic review and meta-analysis, we observed that the use of ABVS devices in TLH for benign indication is associated with a statistically significantly lower intraoperative blood loss and total operative time than conventional bipolar instruments. In contrast, no differences in hospital stay length and complication rate were observed. Only four studies were eligible, and the quality of included studies was low with a high risk of bias, confirming the limited investigation of the energy devices in TLH. Of note, although minimally invasive hysterectomy for benign indications is widely performed, not all surgical steps and technical aspects have been thoroughly investigated in well-conducted studies, and some authors stressed the need to standardize laparoscopic hysterectomy to optimize the procedure in terms of safety and performance [38–40].

ABVS devices are widely used in other surgical specialties and seem to provide substantial clinical advantages. Appendectomy [41], thyroidectomy [42], and hepatic resection [43] were demonstrated to be as safe as conventional instruments with shorter operative time. ABVS devices provided better hemostatic control in abdominoplasty [44], laparoscopic nephrectomy [16], colorectal surgery [13], oral cancer [45], and spinal surgery [46]. On that basis, the American Society of Gastrointestinal and Endoscopic Surgeons (SAGES) stressed the importance of energy knowledge in the operating theatre and developed the FUSE (Fundamental Use of Surgical Energy) education program aiming at the efficient and safe use of surgical energy devices [47]. However, the specific type and difficulty of the surgical procedure have an important impact on the possible advantages provided by the use of ABVS devices, and the clinical utility of these instruments must be confirmed in TLH.

Based on the present systematic review and meta-analysis results, the observed blood loss reduction in the ABVS devices group was small (39 mL; *p* = 0.004) and probably clinically irrelevant, impeding support for their regular use in every TLH to reduce blood loss. Moreover, proposed methods to estimate blood loss were various and could suffer from inaccuracies of collection, quantification, and intra and interobserver variability, especially for small quantities. Indeed, it must be noted that included studies focused on normal-size uteri (mean size ranges from 126 to 465 gr) and excluded very large uteri or other complex hysterectomies [4]. In selected cases, even a small reduction in total blood loss could be considered helpful, and a greater clinical advantage could be obtained by combining different techniques for reducing blood loss, such as ABVS devices and uterine artery closure at the origin [39]. Therefore, the lower intraoperative blood loss associated with ABVS devices may be higher and more clinically relevant in selected patients.

Regarding total operative time, the observed reduction of 8 min did not allow us to suggest an economic advantage provided using ABVS devices in TLH. Considering the average cost of a standard operative room (5 Euro/minute) [5, 34] and the higher cost of an ABVS device compared to conventional bipolar instruments, we could estimate the need for a 20–30 min reduction in total operative time to balance the increased expense [5, 37]. One included study reported data stratified per learning curve [26]. Based on observed differences, authors speculated on a greater reduction in total operative time using ABVS devices after adequate training, further supporting the potential economic sustainability of ABVS devices if used by an expert surgeon in selected patients [26].

Notwithstanding any result supporting or discouraging the use of ABVS devices in TLH, this systematic review is limited by the quality of included studies, which is low mainly due to unclear allocation concealment, lack of blinding, and inaccuracy of reported outcomes. The number of included studies and pooled patients was limited, determining a sample size underpowered to identify events with low frequency. Although the total intraoperative complication rate did not differ between ABVS devices and conventional bipolar energy instruments, the effect on post-operative complications remains unclear. The limited number of studies impedes addressing factors, such as the surgeon’s experience and technical differences between devices, whether ABVS or conventional bipolar electrosurgery. In this regard, ABVS devices share many aspects concerning the use of bipolar energy and feedback systems. Still, each model is characterized by specific features potentially impacting surgical performance and related outcomes. A RCT by Aytan et al. [25] did not demonstrate differences in terms of blood loss, total operative time, or other outcomes between Ligasure and PKS in benign hysterectomy; therefore, in our meta-analysis, we included them in a common ABVS device group, although this may represent a limitation. Finally, there was no evidence to determine the impact on mortality, surgeon satisfaction, quality of life, costs, and potential advantages of robotic surgery. The adoption of a solid evidence-based approach in performing this systematic review and meta-analysis is a strength of our study that allows us to highlight the lack of evidence. We did not include TLH performed for malignant indications to preserve a homogenous population among pooled studies. Nonetheless, considering the two studies which were excluded due to malignant indications by Lee et al. [35] and Fagotti et al. [36], a statistically significant reduction in total blood loss (*p* = 0.03) and a shorter total operative time (*p* = 0.001) in the ABVS device group was reported, which is consistent with our findings.

In conclusion, although our findings showed that ABVS devices reduce blood loss and operative time in TLH without increasing related complications, the available evidence is limited and cannot support their routine use during this surgical procedure due to unproven clinical relevance. Nevertheless, a higher level of evidence on larger groups of patients is needed before providing definitive recommendations on ABVS devices for TLH. In this regard, future well-designed studies should improve methodology and outcomes reporting. The method for blood loss estimation must be extensively described and should include mathematical models based on pre- and post-operative biochemical parameters in addition to intraoperative estimated blood loss. Moreover, operative time should be provided in all its components, with particular attention to the hysterectomy time from the beginning of round ligament transection to colpotomy. Hysterectomy time is more informative than total operative time, allowing addressing for additional surgical procedures, particularly in observational studies, such as adhesiolysis or salpingectomy/adnexectomy. Regarding the significant cost gap between ABVS devices and conventional bipolar instruments, a detailed cost analysis should be included in future reports to guide clinical practice effectively. Finally, further research specifically focused on those cases that could benefit more from ABVS devices during minimally invasive hysterectomy is highly recommended.

## Data Availability

The data that support the findings of this study are available from the corresponding author upon reasonable request.
